# Transgelin-2 in B-Cells Controls T-Cell Activation by Stabilizing T Cell - B Cell Conjugates

**DOI:** 10.1371/journal.pone.0156429

**Published:** 2016-05-27

**Authors:** Bo-Ra Na, Min-Sung Kwon, Myoung-Won Chae, Hye-Ran Kim, Chang-Hyun Kim, Chang-Duk Jun, Zee-Yong Park

**Affiliations:** 1 School of Life Sciences, Immune Synapse Research Center and Cell Dynamics Research Center, Gwangju Institute of Science and Technology, Gwangju, Korea; 2 School of Life Sciences, Gwangju Institute of Science and Technology, Gwangju, Korea; 3 World Institute of Kimchi, Gwangju, Korea; University Paris Sud, FRANCE

## Abstract

The immunological synapse (IS), a dynamic and organized junction between T-cells and antigen presenting cells (APCs), is critical for initiating adaptive immunity. The actin cytoskeleton plays a major role in T-cell reorganization during IS formation, and we previously reported that transgelin-2, an actin-binding protein expressed in T-cells, stabilizes cortical F-actin, promoting T-cell activation in response to antigen stimulation. Transgelin-2 is also highly expressed in B-cells, although no specific function has been reported. In this study, we found that deficiency in transgelin-2 (*TAGLN2*^*-/-*^) in B-cells had little effect on B-cell development and activation, as measured by the expression of CD69, MHC class II molecules, and CD80/86. Nevertheless, in B-cells, transgelin-2 accumulated in the IS during the interaction with T-cells. These results led us to hypothesize that transgelin-2 may also be involved in IS stability in B-cells, thereby influencing T-cell function. Notably, we found that transgelin-2 deficiency in B-cells reduced T-cell activation, as determined by the release of IL-2 and interferon-γ and the expression of CD69. Furthermore, the reduced T-cell activation was correlated with reduced B-cell–T-cell conjugate formation. Collectively, these results suggest that actin stability in B-cells during IS formation is critical for the initiation of adaptive T-cell immunity.

## Introduction

Activation of T-cells requires cell-to-cell contact with antigen presenting cells (APCs) [1]. The formation of the immunological synapse (IS) at the interface between the T-cell and APC leads to a complex reorganization of surface and intracellular molecules. Signal-dependent rearrangements in the actin cytoskeleton help sustain correct temporal and spatial control of the activation process [1]. Over the last decade, the body of knowledge regarding this reorganization process has grown rapidly, adding new players and providing insight into the mechanisms of T-cell biology. The knockout/knockdown or chemical inhibition of specific actin-regulating proteins, such as Arp2/3, WASp, WAVE2, HS-1, cofilin, and L-plastin, have distinct effects on T-cell migration, activation, and proliferation [2,3]. In contrast to T-cells, however, the functions of actin-reorganizing proteins in APCs are relatively unknown. Moreover, it remains to be elucidated whether and how actin-controlling proteins in APCs affect T-cell function.

We recently reported that transgelin-2, a 22 kDa actin-binding protein, stabilizes cortical F-actin and thereby maintains the F-actin content at the IS, allowing T-cell activation following T-cell receptor (TCR) stimulation [4]. The transgelin family of actin cross-linking/gelling proteins comprises three isoforms that share 80% homology: transgelin-1 (highly expressed in smooth muscle cells [5]), transgelin-2 (associated with various types of cancers [6,7]), and transgelin-3 (abundant in brain tissues [8]). Each transgelin protein contains an N-terminal calponin homology (CH) domain and a C-terminal calponin-like module (CLIK^23^) domain [9,10]. The best-characterized isoform, transgelin-1, is an abundant smooth muscle–specific protein that serves as a marker of smooth muscle tissue. Transgelin-3 (also known as neuronal protein 22, NP22, or NP25) is specifically expressed in brain tissue and is upregulated in the superior frontal cortex and hippocampus in alcoholic humans and rat models of alcoholism [11,12]. In contrast to the other members, the biology of transgelin-2 is poorly understood, but it is known to be the only isoform expressed in B-cells.

In B-cells, actin dynamics are essential for the clustering and capping of B-cell antigen receptors (BCRs) and the amplification of intracellular signaling cascades [13,14]. They are also important for antigen uptake via BCR internalization and antigen trafficking into the late endosome [15,16]. Thus, many actin-regulating or -controlling proteins are involved in BCR-related signaling events. For example, Syk-dependent actin dynamics regulate endocytic trafficking and antigen processing [17]. Btk regulates BCR-mediated antigen processing and presentation by controlling the actin cytoskeleton [18]. Actin-binding protein 1 also regulates BCR signaling and antigen processing and presentation by actin cytoskeleton rearrangement [16]. Additionally, cofilin-mediated actin severing controls B-cell spreading and BCR microcluster formation [19]. Because transgelin-2 stabilizes the actin cytoskeleton by antagonizing cofilin function in T-cells [4], we sought to determine the function of transgelin-2 in actin stability in B-cell biology.

In this study, we found that transgelin-2-knockout (*TAGLN2*^*-/-*^) mice exhibit normal B-cell development, as determined by the proportions of B-cell subpopulations and the surface expression of certain markers. In addition, *TAGLN2*^*-/-*^ B-cells had no difference in the expression of activation makers such as CD69, MHC class II molecules, and CD80/86. However, we found that transgelin-2 in B-cells is necessary for the proper stabilization of T cell—B cell conjugates. *TAGLN2*^*-/-*^ B-cells could not support proper adhesion to T-cells and did not properly activate T-cells after conjugating with them. Our results suggest that actin cytoskeleton in B-cells is crucial for regulation of T-cell activation through stabilizing T-cell and B-cell conjugates.

## Materials and Methods

### Reagents and antibodies

Lipopolysaccharide (LPS), poly-L-lysine (PLL), phorbol 12-myristate 13-acetate (PMA), and ionomycin were obtained from Sigma-Aldrich (St. Louis, MO). Goat polyclonal anti-mouse IgM antibodies were purchased from Jackson Immunoresearch Laboratories (West Grove, PA). Mouse IL-4 was obtained from Peprotech (Rocky Hill, NJ). Anti-CD40 antibody was purchased from BD PharMingen (San Diego, CA). *Staphylococcus* Enterotoxin E and B (SEE and SEB) were purchased from Toxin Technology (Sarasota, FL). OVA 323–339 peptides were purchased from InvivoGen (San Diego, CA). Life Technologies (Waltham, MA) supplied 5-(and-6)-(((4-chloromethyl)benzoyl)amino)tetramethylrhodamine (CMTMR) and 5-chloromethylfluorescein diacetate (CMFDA). Rabbit polyclonal anti-transgelin-2 antibodies were generated as previously described [4]. Rabbit polyclonal anti-transgelin-1 was purchased from Santa Cruz Biotechnology (Dallas, TX). Mouse monoclonal anti-transgelin-3 was purchased from Abcam (Cambridge, MA). Rabbit polyclonal anti-β-actin, horseradish peroxidase-conjugated anti-mouse IgG, and anti-rabbit IgG were obtained from Cell Signaling Technology (Danvers, MA). Phycoerythrin (PE)-conjugated antibodies for mouse CD19, CD23, CD43, CD69, MHCII, CD80, CD86, and IgM were purchased from eBioscience (San Diego, CA). Allophycocyanin (APC)-conjugated anti-mouse B220 antibodies and fluorescein isothiocyanate (FITC)-conjugated antibodies for mouse MHCII and CD4 were also purchased from eBioscience. Peridinin-chlorophyll proteins (PerCP)-Cy5.5 conjugated antibodies against mouse IgD, CD21, and CD25 were purchased from Biolegend (San Diego, CA).

### Cells

Jurkat (TIB-152; ATCC, Manassas, VA), Raji B (CCL-86; ATCC), A20 (TIB-208; ATCC), and A7r5 (CRL-1444; ATCC) cell lines were maintained in RPMI 1640 medium or DMEM medium (GIBCO/Invitrogen, Waltham, MA) supplemented with 10% (vol/vol) FBS (GIBCO/Invitrogen), 100 U/ml penicillin (GIBCO/Invitrogen), and 100 mg/ml streptomycin (GIBCO/Invitrogen). After obtaining written informed consent, human primary PBLs were isolated from healthy donors by dextran cosedimentation and centrifugation through a discontinuous Ficoll gradient (GE healthcare, Pittsburgh, PA). Human CD3^+^ and CD19^+^ cells were isolated from PBLs using MACS cell separation (Miltenyi Biotec, San Diego, CA). All experiments using human PBLs were approved by the Ethics Committee of the School of Life Sciences, Gwangju Institute of Science and Technology (GIST). Mouse CD3+ T cells were purified from dispersed spleen and lymph node cells using a T cell enrichment column (R&D Systems, Minneapolis, MN), and B cells were purified using a Mouse B cell enrichment kit (STEMCELL Technologies, Canada). Mouse cells were maintained in RPMI 1640 medium supplemented with 10% FBS, 100 U/ml penicillin, 100 mg/ml streptomycin, 1× MEM non-essential amino acid (GIBCO/Invitrogen), 1 mM sodium pyruvate (GIBCO/Invitrogen), and 50 μM 2-Mercaptoethanol (Sigma). The purity of each cell population was confirmed to be >95% by flow cytometry. All cells were cultured in a humidified 5% CO_2_ incubator at 37°C.

### Mice

C57BL/6 wild-type mice were obtained from Damul Science (Korea). For generation of TAGLN2 knockout mice, murine genomic DNA for *TAGLN2* was obtained from 129/SvJ mouse J1 embryonic stem (ES) cells by PCR. A targeting vector was constructed to delete nucleotides 14,691–15,479 containing exon 2 of *TAGLN2* using a long arm fragment and two short arm fragments ligated into the pOSDupDel.Neo vector. The targeting vector was then electroporated into 129/SvJ ES cells after linearization using *Not*I. Positive recombinant ES cells were identified using PCR and then microinjected into C57BL/6 blastocysts. The resulting chimeric mice were then mated with C57BL/6 mice. Germline transmission was confirmed using Southern blot analysis (Macrogen, Korea). Interbreeding of the heterozygous mice was performed to generate homozygous TAGLN2-deficient mice. OVA-specific TCR transgenic line II (OTII) mice were used, which contain transgenic inserts for mouse α-chain and β-chain TCR subunits specific for OVA 323–339 in the context of I-A^b^ (Jackson Laboratory, Bar Harbor, ME). The same number of male and female mice that were age-matched (8–10 weeks old) were used for experiments. All mice were housed in specific-pathogen-free conditions on a 12:12 light-dark cycle, and food and water were available *ad libitum*. Mice were sacrificed by cervical dislocation, and all efforts were made to minimize suffering. All mouse experiments were performed in accordance with protocols approved by the Animal Care and Use Committee of the School of Life Sciences, Gwangju Institute of Science and Technology (GIST).

### Plasmids

To generate a transgelin-2 construct, a human transgelin-2 clone coding the full-length open reading frame was purchased from ImaGenes GmbH (Germany). The human transgelin-2 was sub-cloned into the pEGFP_N1 vector and pHJ-1 vector as a lentiviral vector.

### Lentiviral infection and cell transfection

To establish a stable cell line, cDNA from the pHJ-1 vector was cotransfected with the lentiviral packaging vectors into HEK293T cells (CRL-1573; ATCC) using Lipofectamine 2000 (Life Technologies). After 48 h, the supernatants were collected and used to infect Raji B-cells and Jurkat T-cells by centrifugation at 800 g for 90 min in the presence of 8 μg/ml Polybrene. Transient transfection of the LifeAct_mCherry vector into Raji B-cells and Jurkat T-cells was performed by Amaxa technology using nucleofector kit V (Lonza, Allendale, NJ).

### B-cell and T-cell stimulation

Mouse B-cells were stimulated with LPS (50 μg/ml), goat anti-IgM antibody (10 μg/ml), PMA (200 nM) and ionomycin (2 μM), or IL-4 (1 μg/ml) and anti-CD40 (2 μg/ml) for the indicated times. In some cases, Raji B-cells were stimulated with 10 μg/ml LPS. For superantigen stimulation, mouse CD4^+^ T-cells were incubated with 5 μg/ml SEB-pulsed mouse B-cells. For OVA stimulation, CD4^+^ T-cells isolated from OTII mice were cultured with mouse B-cells pulsed with 10 μg/ml OVA 323–339 for the indicated times.

### Western blot analysis

Cells or the homogenized brain tissue from a C57BL/6 mouse were lysed in ice-cold lysis buffer (50 mM Tris-HCl, pH 7.4, containing 150 mM NaCl, 1% Triton X-100, and one tablet of Complete protease inhibitors) for 1 h on ice. Lysates were centrifuged at 16,000 g for 30 min at 4°C, and the supernatant was eluted with SDS sample buffer (100 mM Tris-HCl, pH 6.8, 4% SDS, and 20% glycerol with bromophenol blue) and heated for 5 min. The proteins were separated through 12% SDS-PAGE gels and were transferred to a nitrocellulose membrane by means of a Trans-Blot SD semidry transfer cell (Bio-Rad, Hercules, CA). The membrane was blocked in 5% skim milk (1 h), rinsed, and incubated overnight with the intended antibodies in TBS containing 0.1% Tween 20 (TBS-T) and 3% skim milk. Excess primary antibody was then removed by washing the membrane four times in TBS-T. The membrane was then incubated with peroxidase-labeled secondary antibodies (0.1 μg/ml, anti-rabbit or anti-mouse) for 1.5 h. After three washes with TBS-T, bands were visualized by western blot (reagents from Intron Biotechnology, Korea) and exposed to X-ray film.

### Detection of cell surface proteins

For flow cytometry analyses of cell surface protein expression, 5 × 10^4^ cells per sample were washed with 500 μl of FACS buffer (2% FBS/PBS), resuspended in 50 μl of antibody staining solution (1 μg/ml fluorescent-conjugated antibodies in FACS buffer) for 15 min at room temperature in the dark, and then washed. The cells were fixed with 2% paraformaldehyde/PBS for 40 min at 4°C and washed with FACS buffer. Data were acquired on a FACSCanto (BD Biosciences, San Jose, CA) and analyzed with FlowJo software (Tree Star, Ashland, OR).

### Confocal microscopy and imaging analysis

GFP–Raji B-cells and transgelin-2_GFP–Raji B-cells were transiently transfected with LifeAct_mCherry by Amaxa (Lonza) and plated on coverslips (18 mm diameter; Marienfeld, Germany) coated with PLL. After 10 min of preincubation, LifeAct-positive cells were imaged in imaging medium (137 mM NaCl, 5 mM KCl, 1 mM sodium phosphate, 6 mM D-glucose, 1 mM CaCl_2_, 0.5 mM MgCl_2_, 1% BSA, and 10 mM HEPES, pH 7.3) at 37°C using a 100×, NA 1.40 oil immersion objective lens and a laser-scanning confocal microscope (FV1000; Olympus, Japan). To test for transgelin-2 and F-actin accumulation at B-cell–T-cell contact sites, SEE-loaded Raji B-cells were mixed with T-cells and preincubated in solution for 10 min. After incubation, conjugated cells were placed on PLL-coated coverslips for 10 min and imaged at 37°C. The data were obtained, processed, and analyzed by FLUOVIEW software (Olympus). For the 3D localization assay, z stacks were generated from optical sections taken at 0.2 μm intervals, and FLUOVIEW software was used for 3D image reconstruction. The fluorescence intensity profiles of TG2_GFP and LifeAct_mCherry in the cells along the line (a–b) or at the plane of contact site reconstructed by 3D imaging along the line (c–d) and Pearson’s correlation coefficients (r), measuring the colocalization, were also measured and calculated using the FLUOVIEW software.

Redistribution of TG2_GFP or GFP at the contact site in the B-cells or T-cells during IS formation was calculated as the ratio between the mean fluorescence intensity of GFP in the half portion of contact site (C) and opposite site (O) in the cells. At least 50 conjugates were counted in each of three independent experiments. All the data were plotted using KaleidaGraph (Synergy Software, Reading, PA).

### Measuring the secretion of cytokines

Splenic CD3^+^ T-cells and CD4^+^ T-cells isolated from C57BL/6 and OTII mice, respectively, were stimulated by B-cells as described in “B-cell and T-cell stimulation,” above. After 24 h, the amount of IL-2 and IFN-γ in the supernatants was determined by ELISA with DuoSet Mouse ELISA kits (R&D Systems).

### Conjugation assay

Mouse CD3^+^ T-cells and B-cells were stained with CellTracker Green CMFDA and Orange CMTMR, respectively (Life Technologies). B-cells were incubated with 5 μg/ml SEB or vehicle control for 30 min at 37°C, washed, and resuspended in mouse cell medium. For conjugation, equal numbers of B-cells and T-cells were mixed and incubated at 37°C for 30 min. The relative proportion of orange, green, and orange-green events in each tube was determined by two-color flow cytometry using a FACSCanto and analyzed with FlowJo software. The number of gated events counted per sample was at least 10,000. The percentage of conjugated T-cells was determined as the number of dual-labeled (CMFDA- and CMTMR-positive) events divided by the number of CMFDA-positive T-cells. CD4^+^ T-cells isolated from OTII mice and OVA 323–339 peptide-loaded B-cell conjugates were also stained, incubated, and determined as described above.

### Statistics

Mean values were calculated using data from three independent experiments conducted on different days. Unpaired Student’s t tests and one-way analysis of variance tests were used to determine significance. Differences between groups were considered significant at *p < 0*.*05*.

## Results

### Transgelin-2 is expressed in B-cells

**Fig 1:**
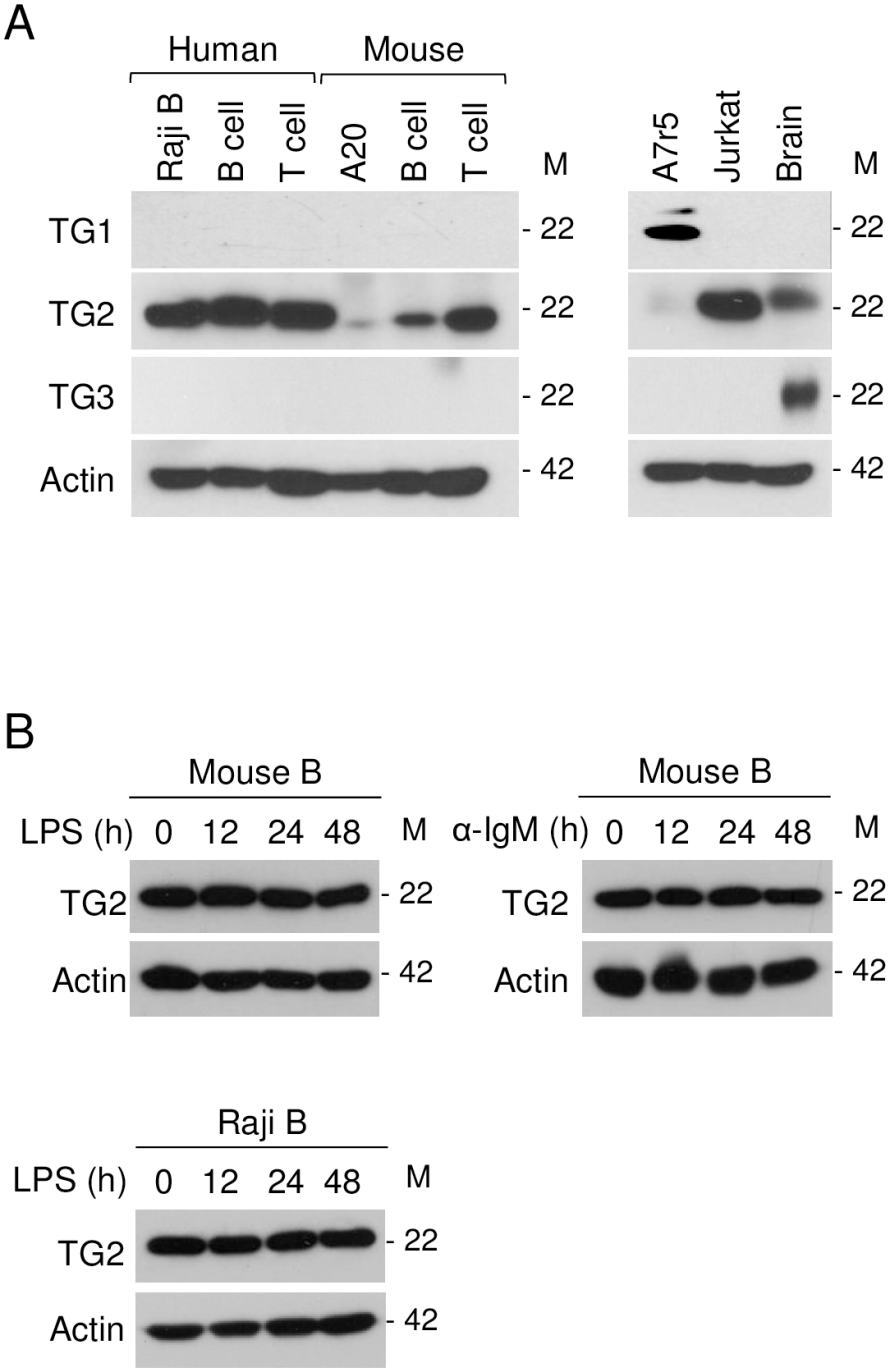
Transgelin-2 is expressed in B-cells. (A) Western blot analysis of transgelin (TG)-1, -2, and -3 expression in the indicated cells. Raji B and A20 are a human and mouse B-cell line, respectively. Human B-cells and T-cells were isolated from human PBL, and mouse B-cells and T-cells were purified from mouse splenocytes. Lysates from A7r5, Jurkat cells, and brain tissue were used as positive controls for transgelin-1, -2, and -3. (B) Mouse B-cells and human B-cells were stimulated with LPS (10 μg/ml) or anti-mouse IgM (10 μg/ml) for the indicated time. Transgelin-2 expression was detected by western blot. All data shown are representative of three independent experiments. M denotes molecular mass.

In a previous study, we found that both CD3^+^ T- and CD19^+^ B-cells express transgelin-2 [4]. To confirm its expression in relation to the other transgelin isoforms, we performed a western blot and found that purified primary T- and B-cells, including cell lines, expressed only transgelin-2 (Fig 1). As positive controls, the aortic smooth muscle cell line A7r5, which expresses transgelin-1, and brain tissues, which express transgelin-2 and -3, were used (Fig 1). We next determined whether transgelin-2 expression is regulated by stimulation. We found no change in protein levels in response to the stimuli, LPS and anti-IgM antibody (Fig 1), suggesting that transgelin-2 expression in B-cells is not altered by extrinsic factors.

### Transgelin-2 knockout (*TAGLN2*^*-/-*^) does not significantly affect B-cell development

**Fig 2:**
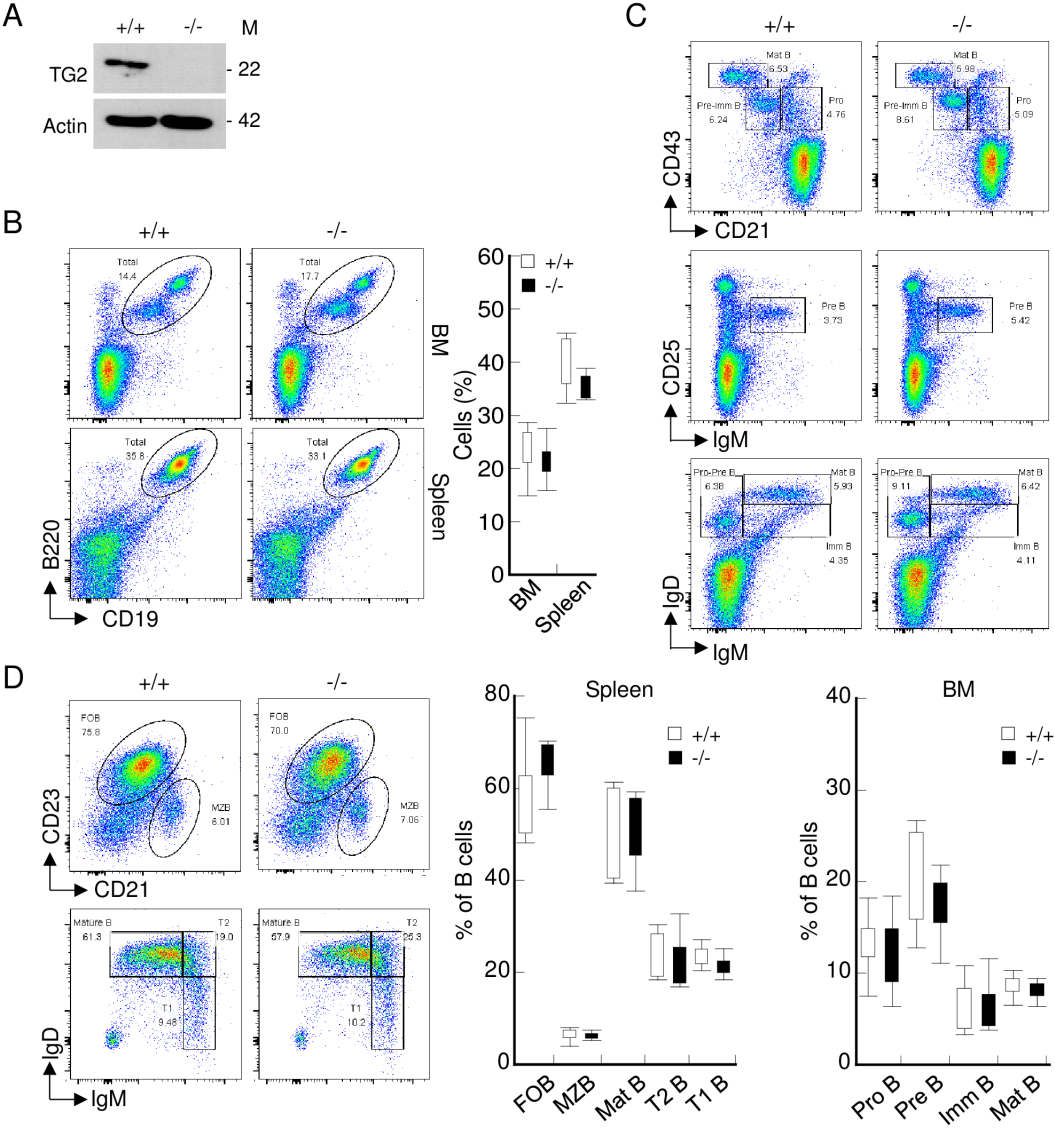
Transgelin-2-knockout mice exhibit normal B-cell development. (A) Expression of transgelin-2 in B-cells obtained from *TAGLN2*^*+/+*^ (+/+) and *TAGLN2*^*-/-*^ (-/-) mice was determined by western blot analysis. M denotes molecular mass. (B) Single cell suspensions of bone marrow (BM) and spleen were tested for the presence of CD19^+^ and B220^+^ cells. The numbers in the dot plots indicate the percentage of CD19^+^B220^+^ B-cells. The mean percentages of positive cells in BM and spleen are shown in the graph (right). (C) Single cell suspensions of BM from *TAGLN2*^*+/+*^ and *TAGLN2*^*-/-*^ were assessed for the expression of CD43, CD21, CD25, IgM, and IgD by flow cytometry. The numbers in the dot plots indicate the percentage of B-cell subsets within the respective gates. The graph shows the average percentage of indicated B-cell development states in the BM cells (bottom). (D) Single cell suspensions of spleen from *TAGLN2*^*+/+*^ and *TAGLN2*^*-/-*^ cells were pre-gated on B220, and expression of CD23, CD21, IgM, and IgD was assessed by flow cytometry. The numbers in the dot plots indicate the percentage of B-cell subsets within the respective gates. The graphs show the average percentage of the indicated B-cell subset in the B220^+^ B-cells gate (right). All dot plots are representative of two experiments with five mice, and the bar graphs are shown as means ± SD of two experiments with five mice each.

We previously found that transgelin-2 deficiency does not change the sizes of the primary and secondary lymphoid organs [4]. To test whether transgelin-2 knockout affects B-cell immunity, we investigated the B-cell subpopulations and surface expression of B-cell markers. The absence of transgelin-2 expression was confirmed by western blot analysis (Fig 2). The normal proportions of B-cell subpopulations were observed in the BM and spleen of *TAGLN*^*-/-*^ mice (Fig 2).

### Transgelin-2 knockout had little effect on B-cell functions

**Fig 3:**
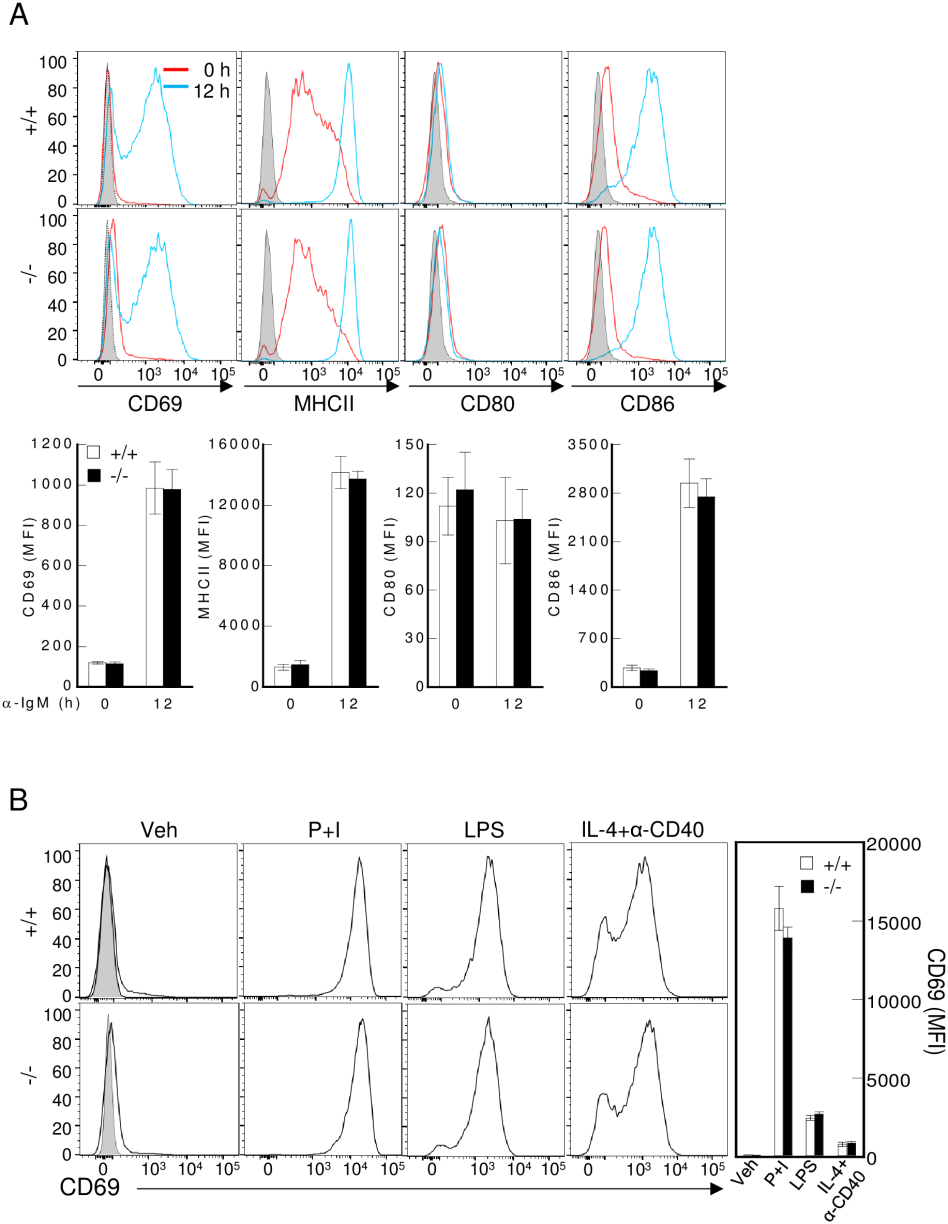
Transgelin-2 knockout had little effect on B-cell function. (A) Splenic B-cells obtained from *TAGLN2*^*+/+*^ and *TAGLN2*^*-/-*^ mice were activated with anti-mouse IgM (10 μg/ml) for 12 h, and then CD69, MHCII, CD80, and CD86 expression were determined by flow cytometry. FACS histograms are representatives of three independent experiments, n = 3–6 mice per group. The bar graphs denote average mean fluorescence intensities (MFI) of each proteins ± SD. (B) B-cells were activated by PMA (200 nM) and ionomycin (1 μM), LPS (10 μg/ml), or IL-4 (1 μg/ml) and anti-CD40 (2 μg/ml). After 12 h, the CD69 expression level was assessed by flow cytometry (black line). FACS histograms are representatives of three independent experiments, n = 5 mice per group. The bar graph denotes average mean fluorescence intensities (MFI) ± SD. Gray shading shows isotype control. Veh, vehicle.

We next tested whether transgelin-2 knockout affects the function of B-cells. CD69 is a transmembrane C-type lectin protein that is induced by the activation of lymphocytes [20]. MHC class II is expressed on professional APCs and supports CD4^+^ T-cell activation by displaying antigens [21,22]. CD80 and CD86 are expressed on APCs and bind CD28, stimulating T-cell activation [23]. The upregulation of these molecules is a hallmark of B-cell activation, and we measured their expression after B-cell stimulation (wild-type and transgelin-2-knockout B-cells) with various stimuli including anti-IgM, PMA plus ionomycin, and LPS plus anti-CD40. However, no significant changes in surface expression were detected by flow cytometry in transgelin-2-knockout B-cells as compared with the wild type (Fig 3), suggesting that transgelin-2 has little effect on B-cell activation.

### Transgelin-2 in B-cells accumulates at the IS

**Fig 4:**
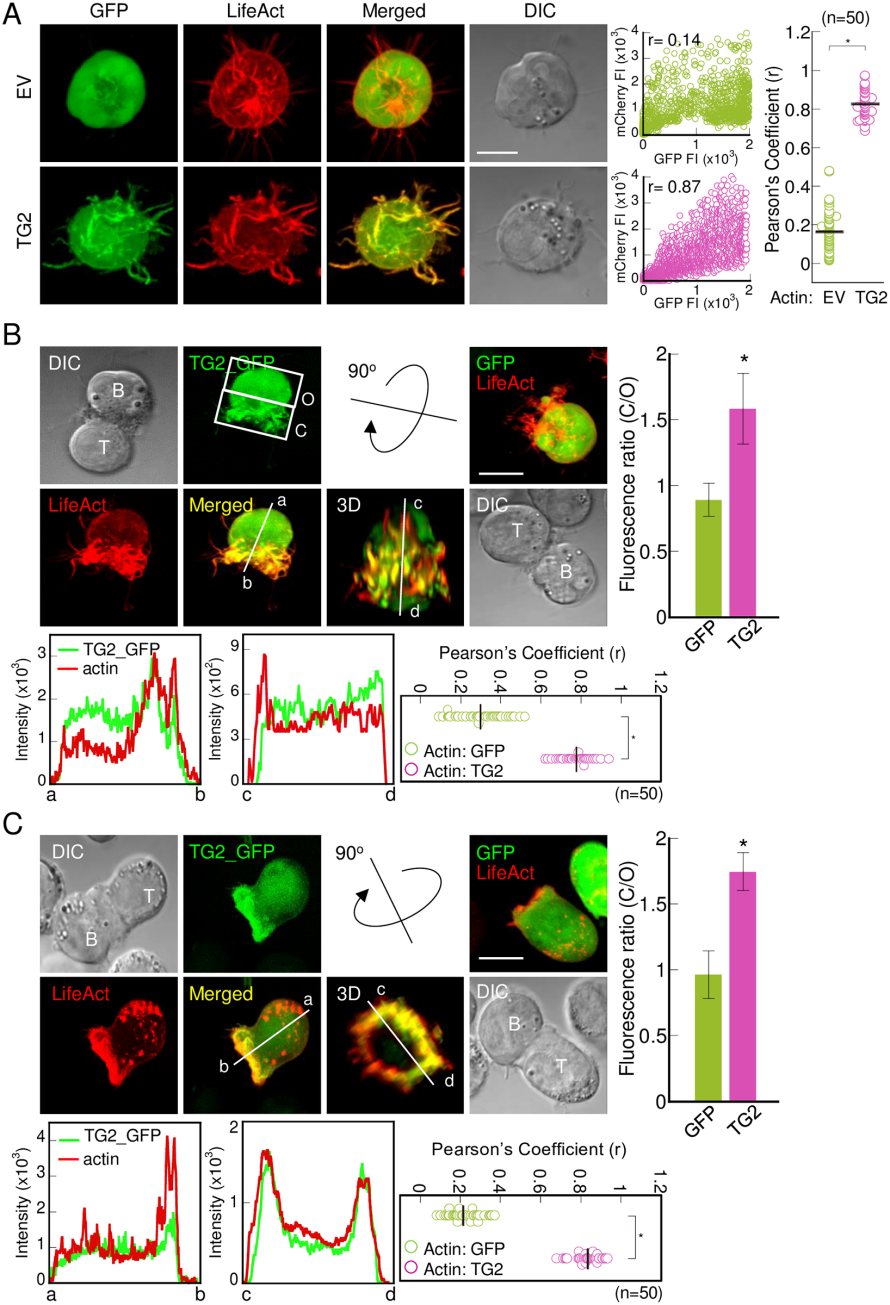
Transgelin-2 in B-cells colocalizes with F-actin and accumulates at the IS. (A) GFP–Raji B (EV) cells and TG2_GFP–Raji B (TG2) cells transfected with LifeAct_mCherry on PLL-coated coverslips were imaged. The colocalization of GFP and mCherry signals was estimated by the Pearson’s correlation coefficient (r) (middle). r values of single cells are represented single dots, and more than 50 cells were examined. Horizontal bars indicates the means (right).* *p* < 0.05 versus GFP-Raji B cells. (B) SEE-loaded TG2_GFP–Raji B cells (left) and GFP–Raji B cells (right) were pre-incubated for 10 min with Jurkat T cells and placed on PLL-coated coverslips. Transgelin-2 (green) and LifeAct (red) redistribution at the contact site of the IS were imaged. Reconstitution of the 3D view of the T cell–B cell contact site and analysis of fluorescence intensity profiles were performed using FLUOVIEW (bottom left and middle). The r values for GFP or TG2_GFP and actin in images of Raji B cells after IS formation were calculated. Each dot represents a single cell and vertical bars indicate the means (bottom right). GFP or TG2_GFP redistribution were estimated as described in Materials and Methods (right). * *p* < 0.05 versus GFP-Raji B cells. (C) TG2_GFP-Jurkat T cells and GFP-Jurkat T cells were pre-incubated for 10 min with SEE-loaded Raji B cells and placed on PLL-coated coverslips. The cells were imaged and analyzed as described in (B). * *p* < 0.05 versus GFP-Jurkat T cells B cells. All images are representative of more than 50 cells examined from three independent experiments. DIC, differential interference contrast. Bars, 10 μm.

We previously demonstrated that transgelin-2 localizes to the distal region of the IS (distal-supramolecular activation cluster, d-SMAC) in T-cells [4]. In this study, we determined the location of transgelin-2 in B-cells before and after IS formation. Transgelin-2 was colocalized with the F-actin-rich region in B-cells, as determined by imaging transgelin-2_GFP (TG2_GFP) (Fig 4). In addition, TG2_GFP moved to the contact region after IS formation (Fig 4). However, the accumulation pattern of transgelin-2 in B-cells was different from that of transgelin-2 in T-cells (Fig 4). In B-cells, transgelin-2 accumulated in the actin-rich filopodia, which point towards the T-cell, at the contact site. Taken together, these data suggest that transgelin-2 in B-cells is involved in the stabilization of F-actin and is necessary for capturing T-cells in order to make a stable IS.

### Transgelin-2 in B-cells affects T-cell function

**Fig 5:**
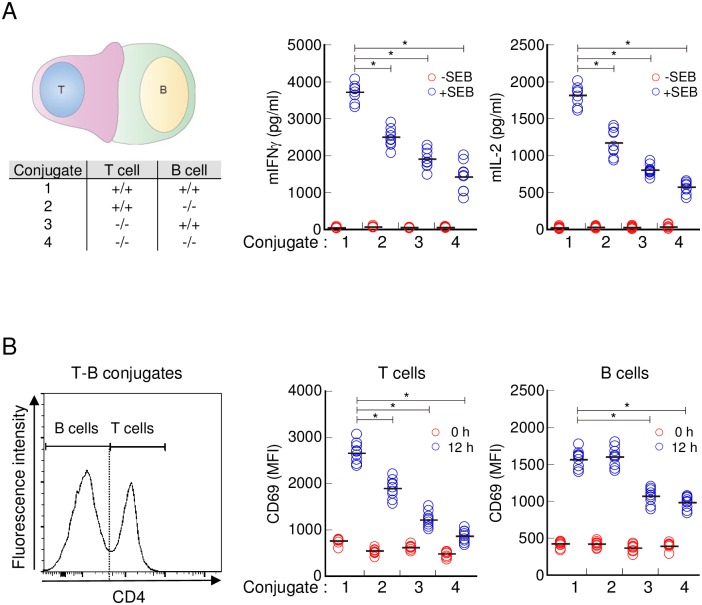
T-cell activation is attenuated by transgelin-2-knockout B-cells during SEB-mediated IS formation. (A) CD4^+^T-cells and B-cells isolated from *TAGLN2*^*+/+*^ and *TAGLN2*^*-/-*^ mice were combined in four configurations to make T-cell–B-cell conjugates (left table). T-cells were incubated with SEB-loaded or -unloaded B-cells for 24 h, and the levels of mIFN-γ and mIL-2 were measured using ELISA. (B) Each conjugate in (A) was incubated for 12 h and stained with FITC-conjugated anti-mouse CD4 and PE-conjugated anti-mouse CD69 antibodies. Conjugates were pre-gated with CD4 expression, and CD69 expression in T-cells (CD4^high^) and B-cells (CD4^low^) was assessed by flow cytometry. Graphs depict cumulative data from three independent experiments with each dot representing an individual mouse (n = 9); bars represent means of all data. * *p* < 0.05 compared with T-cell–B-cell conjugates from TAGLN2^+/+^.

**Fig 6:**
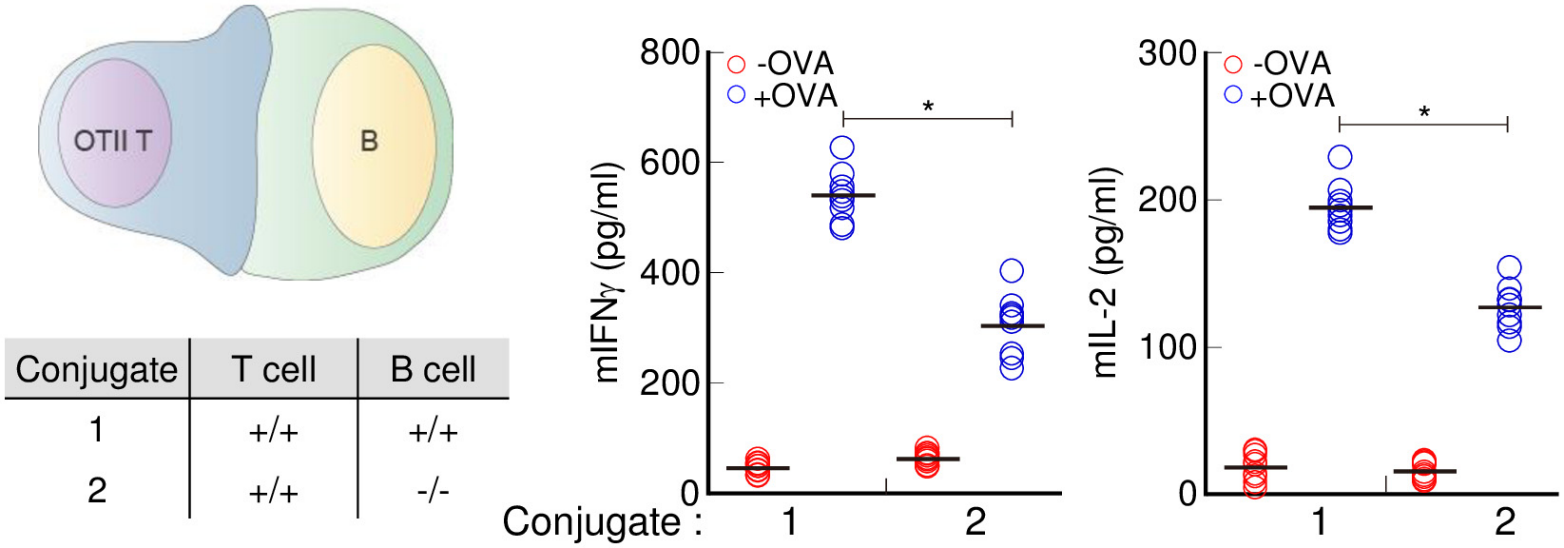
Transgelin-2-knockout B-cells attenuate antigen-specific T-cell activation. CD4^+^ T-cells purified from OTII mice were incubated with OVA-loaded or -unloaded B-cells isolated from *TAGLN2*^*+/+*^ and *TAGLN2*^*-/-*^ mice. Two types of conjugate combinations are displayed in the table (left table). After 24 h of incubation, the levels of mIFN-γ and mIL-2 were measured using ELISA. Graph depicts cumulative data from three independent experiments with each dot representing an individual mouse (n = 9); bars represent means of all data. * *p* < 0.05 compared with B-cells from *TAGLN2*^*+/+*^.

The fact that transgelin-2 in B-ells accumulated at the IS led us to ask whether it could influence T-cell function by stabilizing T-cell–B-cell adhesion. Incubation of *TAGLN2*^*+/+*^ T-cells with *TAGLN2*^*-/-*^ B-cells in the presence of the superantigen SEB decreased T-cell activation relative to that of wild-type B-cells, as determined by cytokine production (mIFN-γ and mIL-2) and CD69 expression, whereas there was no effect on CD69 expression in B-cells (Fig 5). To corroborate the results of the superantigen, we determined the effect of transgelin-2 deficiency in B-cells utilizing T- cells from a transgenic mouse line (OTII) that reacts to ovalbumin (323–339). *TAGLN2*^*-/-*^ B-cells also reduced the mIFN-γ and mIL-2 production in OVA-specific OTII T-cells (Fig 6).

### Transgelin-2 in B-cells participates in enhanced T-cell–B-cell conjugate formation

**Fig 7:**
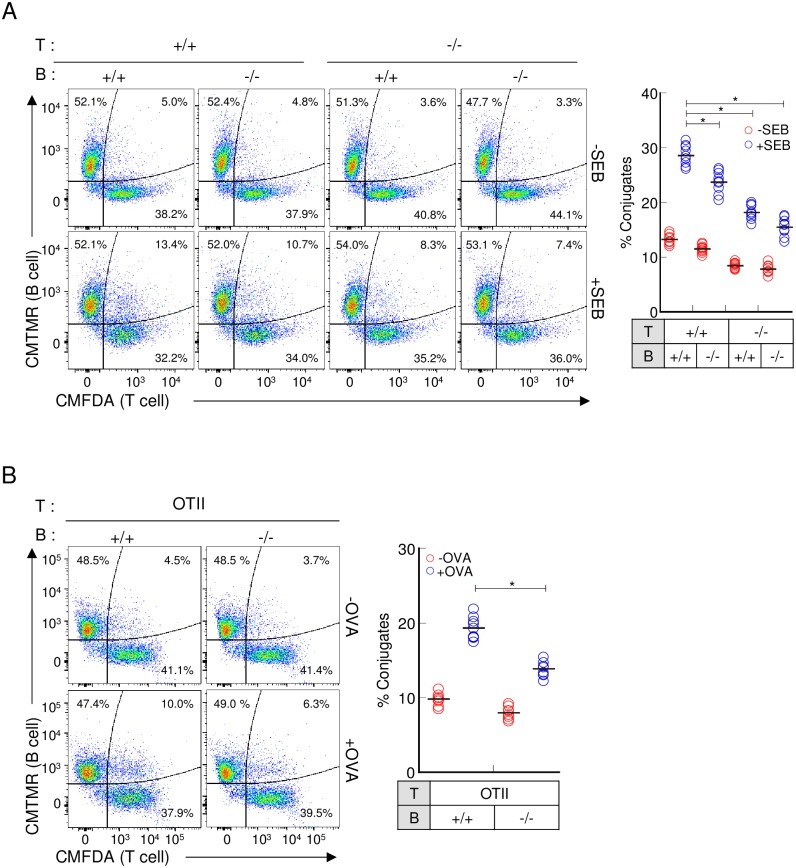
IS formation is reduced in transgelin-2-knockout B-cells. (A) Representative flow cytometry profiles of conjugate formation between T-cells and SEB-loaded or -unloaded B-cells isolated from *TAGLN2*^*+/+*^ and *TAGLN2*^*-/-*^ mice. The percentages of conjugates were calculated as described in Materials and methods. (B) OVA-specific conjugate formation between CD4^+^ T-cells and OVA-loaded B-cells as described in Fig 6, and the percentage of conjugates, determined as in (A). The flow cytometry plots shown are representative of three independent experiments Graphs depict cumulative data from three independent experiments with each dot representing an individual mouse (n = 9); bars represent means of all data. * *p* < 0.05 compared with T-cell–B-cell conjugates from *TAGLN2*^*+/+*^.

To investigate whether the reduced T-cell activation of wild-type T-cells incubated with *TAGLN2*^*-/-*^ B-cells was due to less efficient adhesion between the two cell types, a conjugation assay was performed. Although the percentage of conjugates was less in the preparations with transgelin-2-knockout T-cells, transgelin-2-knockout B-cells also reduced both SEB and OVA-mediated T-cell–B-cell conjugate formation (Fig 7). These results unambiguously indicate that transgelin-2 in B-cells is important for the stabilization of the IS between T-cells and B-cells, and therefore important for T-cell activation.

## Discussion

Transgelin-2 is an actin-binding and actin-stabilizing protein that is highly expressed in both T-cells and B-cells. We previously demonstrated that transgelin-2 in T-cells stabilizes F-actin at the IS, thereby enhancing T-cell activation and effector functions [4]. However, no further work has been reported regarding the function of transgelin-2 in B-cells. In this study, we investigated the function of transgelin-2 in B-cells and found that it plays a role in sustaining the IS, thereby promoting T-cell activation, rather than in B-cell function. Although transgelin-2 in T-cells is more critical for regulation of T-cell activation through actin-mediated intracellular activation signaling as well as stabilization of IS formation [4], our findings show that transgelin-2 in B-cells also plays a certain role in the T-cell immunity.

Northern blot analysis using adult murine tissues revealed that SM22β (*TAGLN2*) is weakly expressed in the spleen and thymus [24]. However, gene expression profiling on human adult tissues demonstrated that *TAGLN2* is predominantly expressed in the human spleen [4]. Previous northern blot analyses of various cell lines demonstrated that EL-4, a mouse T-cell line, also expresses *TAGLN2* [24]. In addition, *TAGLN2* is expressed in lymphocytes, including B-1 cells [25], which is consistent with our results. Together, this indicates that transgelin-2 is mainly expressed in immune tissues and cells. However, transgelin-2 was not specifically up- or downregulated during B-cell activation, suggesting that transgelin-2 is not likely involved in B-cell responses. Nevertheless, the accumulation at the IS suggests that transgelin-2 controls the IS in B-cells, much like it does in T-cells.

The d-SMAC is a peripheral, radial lamellipodium that contains tyrosine phosphatase CD45, dynamic actin, and actin-regulating proteins such as the Arp2/3 complex and cofilin [3,26]. The functions of the d-SMAC in T-cells are relatively well characterized, and it appears to be critical for sustained signaling by allowing the formation of small TCR clusters [27]. However, it is relatively unclear whether APCs also form a typical d-SMAC and whether it works similarly as in T-cells. In addition, molecules in the IS of APCs are not well characterized. Interestingly, there are reports describing that cytoskeleton in dendritic cells has an important role for IS formation and activation of resting T-cells [28,29], demonstrating that actin cytoskeletons are also important for APC side. However, the authors did not address whether APC forms a typical d-SMAC as seen in T-cell side. Our current work unambiguously demonstrates that B-cells do not form a typical d-SMAC during IS formation. Instead, actin and actin regulating protein—transgelin-2—are accumulated in the filopodia, which point towards the T-cell, at the contact site. We therefore suggest that this structure is important for the stabilization of IS as knockout of transgelin-2 significantly reduced T-cell-B-cell conjugate formation. Moreover, we also considered a potential role of transgelin-2 in B-cells for the effective presentation of antigens to T-cells. In this regard, we are currently investigating whether transgelin-2 influences dendritic cell functions in terms of antigen presentation.

During IS formation, activation of CD40 on B-cells by ligation with CD40L on T-cells has an important role for T-cell-dependent B-cell activation [30]. In fact, lowered expression of CD69 on B-cells in conjugation with *TAGLN2*^*-/-*^ T-cells may reflect attenuated CD40-CD40L interaction or CD40-mediated signaling in B-cells. However, normal surface expressions of CD40 on *TAGLN2*^*-/-*^ B-cells and CD40L on *TAGLN2*^*-/-*^ T-cells demonstrate that CD40-CD40L is not involved in CD69 expression in B-cells upon IS formation. Although we do not exactly understand how *TAGLN2*^*-/-*^ T cells affect lowered expression of CD69 in B-cells at present, we can speculate the involvement of integrin (LFA-1) on T-cells, as *TAGLN2*^*-/-*^ T-cells show reduced cell adhesion on ICAM-1 [4] or synapse formation with antigen-loaded B-cells (Fig 7)

Actin is involved in many different cellular processes that are essential for cell growth, differentiation, division, membrane organization, and motility. Mutations or deficiencies in actin-regulating genes encoding WASp, cofilin, actinin, and Vav proteins cause various human diseases [31–34]. Disruption of transgelin-1 promotes inflammation after arterial injury via NF-κB activity [35], and transgelin-1 modulates the phenotype of vascular smooth muscle cells during atherogenesis [36]. Transgelin-1 is also associated with the suppression of cancer metastasis and tumor development [10]. By contrast, transgelin-2 is upregulated in certain tumors [37] and may be involved in tumor development [37]. However, none of the transgelin family members have been reported to be associated with inflammatory diseases. High expression of transgelin-2 in T-cells and B-cells suggests that this small actin-stabilizing protein is involved in immune diseases. Thus, transgelin-2 could be a potential therapeutic target for certain immune disorders including inflammation, hypersensitivity, rheumatoid arthritis, and experimental allergic encephalomyelitis. The current result may also be relevant in the field of T cell immunotherapy. For example, most T cell immunotherapies are focused on controlling T cell functions, which include chimeric antigen receptors [38–40], TCR transgenes [41,42], signaling domains [43], and chemokine receptors [44–46]. Enhancing IS stability by controlling the actin cytoskeletons of APCs may be another goal for T cell immunotherapy.

Proteins that bind the sides of filaments to stabilize F-actin are also involved in actin dynamics. For example, epithelial protein lost in neoplasm (EPLIN) inhibits the branching nucleation of actin filaments through the Arp2/3 complex and promotes the formation of stable actin filament structures such as stress fibers [47]. Bacterial protein SipA, which is required for the efficient entry of *Salmonella typhimurium* into host cells, also stabilizes F-actin [48]. SipA induces extensive polymerization under low-salt conditions, where spontaneous nucleation and polymerization do not occur [49,50]. A striking observation is that, like SipA protein, transgelin family members can extensively polymerize G-actin (data not shown), suggesting that transgelin may have another function in addition to stabilizing F-actin. In this regard, we recently found that transgelin-2 is over-induced in response to LPS in macrophages and that transgelin-2-deficient macrophages have reduced ruffle formation (data not shown). As membrane ruffling in macrophages is important for their phagocytic activity, we are currently investigating whether transgelin-2 is also involved in macrophage function, especially phagocytosis.

In summary, we found that the function of transgelin-2 in B-cells at first appears insignificant when compared with that in T-cells. Nevertheless, a deficiency of transgelin-2 in B-cells resulted in attenuated T-cell function. Although the mechanism by which transgelin-2 in B-cells supports T-cell function needs further study, stabilizing the IS could be a critical mechanism for T-cell function. Future studies with *in vivo* models using intra-vital or multiphoton microscopy will be needed to corroborate our *in vitro* results.
